# The Relationship Between Attentional Capture by Speech and Nonfluent Speech Under Delayed Auditory Feedback: A Pilot Examination of a Dual-Task Using Auditory or Tactile Stimulation

**DOI:** 10.3389/fnhum.2020.00051

**Published:** 2020-02-26

**Authors:** Osamu Ishida, Daichi Iimura, Shoko Miyamoto

**Affiliations:** ^1^Graduate School of Comprehensive Human Sciences, University of Tsukuba, Ibaraki, Japan; ^2^Saitama Municipal Nakamoto Elementary School, Saitama, Japan; ^3^Japan Society for the Promotion of Science, Tokyo, Japan; ^4^Faculty of Human Sciences, University of Tsukuba, Ibaraki, Japan

**Keywords:** sensory modality, speech motor control, attentional capture, involuntary attention shift, communication disorders

## Abstract

Delayed auditory feedback (DAF) leads to nonfluent speech where the voice of a speaker is heard after a delay. Previous studies suggested the involvement of attention to auditory feedback in speech disfluency. To date, there are no studies that have revealed the relationship between attention and nonfluent speech by controlling the attention allocated to the delayed own voice. This study examined these issues under three conditions: a single task where the subject was asked to read aloud under DAF (single DAF task), a dual task where the subject was asked to read aloud while reacting to a pure tone (auditory DAF task), and a dual task where the subject was asked to read aloud while reacting to the vibration of their finger (tactile DAF task). The subjects also performed the single and dual tasks (auditory/tactile) under nonaltered auditory feedback where no delayed voices were involved. Results showed that the nonfluency rate under the auditory DAF task was significantly greater than that under the single DAF task. In contrast, the nonfluency rate under the tactile DAF task was significantly lower compared with that of the single DAF task. Speech became nonfluent when attention was captured by the same modality stimulus, i.e., auditory tone. In contrast, speech became fluent when attention was allocated to the stimulus that is irreverent to auditory modality, i.e., tactile vibration. This indicates that nonfluent speech under DAF is involved in attention capture owing to the delayed own voice.

## Introduction

In day-to-day conversations, we adjust the sound pressure, pitch, and speech speed, among other parameters, based on our auditory feedback. This depends on the environment where speakers are present. Auditory feedback includes air and bone conduction. Air conduction feedback means that the voice is transmitted to a speaker’s own eardrums as air vibrations, and the speaker perceives it. Bone conduction feedback means that the speaker perceives the vibration of their cranial bones caused by articulation. These feedbacks are transmitted *via* the auditory nerve. It is difficult to adjust speech in environments where such auditory feedback is disrupted. In a loud environment, the sound pressure and pitch of the speech increase to compensate for the disruption of auditory feedback (Lombard effect, Siegel and Kennard, 1984).

In delayed auditory feedback (DAF), where the voice of speaker is heard with a delay on the order of a few tens of milliseconds to a few hundred milliseconds, nonfluent speech could occur in the form of word revisions (e.g., yesterday, Mom…Dad), syllable repetitions (e.g., m, m, Mom), or prolongations (e.g., Mo-m). This disfluency effect has been used in previous studies to investigate the relationship between speech and auditory feedback (e.g., Black, 1951; Yates, 1963; MacKay, 1968; Borden, 1979; Siegel et al., 1980; Fukawa et al., 1988; Stuart et al., 2002). In contrast, nonfluent speech occurs less frequently under normal auditory feedback (NAF), where the speaker is heard in real time with no delay. This difference in the occurrence rate of nonfluent speech between DAF and NAF is called the DAF effect (Lee, 1950; Black, 1951). Notably, nonfluent speech significantly increased when feedback was delayed for 200 ms compared with other time delay conditions (100 ms, 400 ms, and 800 ms; Fairbanks, 1955).

Auditory attention to delayed own voice has been suggested to be involved in such nonfluent speech in previous behavioral or neuroimaging studies (MacKay, 1968; Hashimoto and Sakai, 2003; Takaso et al., 2010; Ishida et al., 2019). Nonfluent speech rarely occurs even under DAF when it is difficult to read aloud, such as reading at a fast pace or reading color names that are different from the color of the letters (Stroop task; Fillenbaum, 1963; Zanini et al., 1999). We assume that one cannot pay attention to our speech in these conditions. Therefore, it is considered that speech becomes fluent when attention is focused on a sentence stimulus or speech movement, as this decreases the allocation of attention to DAF (the details have been provided in the “Discussion” section). Furthermore, neuroimaging studies have reported that cerebral blood flow in the superior temporal gyrus, which is involved in auditory attention, significantly increased under DAF compared to NAF (Hirano et al., 1997; Hashimoto and Sakai, 2003; Sakai et al., 2009; Nota et al., 2011); the positive correlation was found between the activity and the frequency of nonfluent speech (Hashimoto and Sakai, 2003; Takaso et al., 2010). These findings could imply the involvement of auditory attention toward nonfluent speech. To date, no studies have been conducted to examine the relationship between attention and nonfluent speech by controlling attention allocation to the delayed own voice under DAF. We assume that forcing the direction of attention toward some stimulus, i.e., DAF, could influence the fluency, and we could realize this situation by performing a dual task.

The phenomenon in which attention is directed to stimuli unrelated to the task, regardless of the subject’s intention, is called attention capture (Theeuwes, 2010). In general, humans can actively allocate attention to and differentiate between stimuli (target stimuli) related to a task. In contrast, when the nontarget stimuli, which are highly salient or novel, or when unexpected stimuli (deviant stimuli) are present, such as in oddball tasks, attention is directed to that stimuli even if it is unrelated to the task (Yantis and Jonides, 1984; Theeuwes, 1991, 1994, 2010; Miller and Buschman, 2013). Attention allocation and capture are also related to task performance. The rate of correct answer improves when attention is allocated to task-related stimuli (Reeves and Sperling, 1986; Cheal and Lyon, 1989). In contrast, distracting effects arise when attention is captured by stimuli unrelated to the task, as the processing involved in the task is interrupted (Schröger and Wolff, 1998). Thus, the effect of attention capture differs depending on whether the disruption stimulus is a task-relevant stimulus.

In a dual task, a subject is asked to perform multiple tasks comprising a primary task and secondary task. According to the capacity theory of attention (Kahneman, 1973), and due to limited attentional resources, the attention resources allocated to each task are considered to be fewer than those allocated to a single task. While the performance decreased in some dual task paradigms compared to single tasks (Appelbaum et al., 2012; Finke et al., 2015; Marsh et al., 2015), performance increased in other dual tasks (Melzer et al., 2001; Swan et al., 2004). It is also reported that, by using the dual task of semantic or phonological similarity (auditory), the performance in a dual task varied between different sensory modalities and a single sensory modality (Salamé and Baddeley, 1982; Hirst and Kalmar, 1987).

We suppose that, under a dual task, nonfluent speech also occurs depending on the deviance of the disruption stimulus (i.e., unimodal/task-relevant or bimodal/task-irrelevant). We set a dual task paradigm in which subjects were asked to read aloud under DAF (this is a single task for control) or read aloud under DAF while reacting to auditory stimulation (unimodal, the modality of the concurrent task was relevant to the primary task) or tactile stimulation (cross-modal, the modality of the concurrent task was irrelevant to the primary task). We hypothesized that speech would become nonfluent when attention was allocated to the own voice under DAF (unimodal task), while nonfluent speech would become attenuated when attention was allocated to sensory feedback rather than to the own voice (cross-modal task).

There are also individual differences regarding the DAF effect (Umeo and Ichinose, 2008; Nota et al., 2011; Chon et al., 2012). As a significant positive correlation between the activity of the superior temporal gyrus and the frequency of nonfluent speech was reported (Hashimoto and Sakai, 2003; Sakai et al., 2009), auditory attention to the delayed own voice could also be explained in the context of individual differences in the DAF effect. Therefore, we hypothesized that speech fluency would increase for speakers who were capable of allocating attention to sensory feedback. We also investigated the relationship between the DAF effect and individual differences of the DAF effect.

## Subjects

We initially conducted an experiment for 22 healthy adults (14 men, six women; average age: 25.20 ± 7.42 years old, and schooling history: 15.95 ± 1.15 years). We calculated the sample size required for the present experiment (power = 0.8, *α* = 0.05, *d* = 0.5; medium effect, i.e., Cohen, 1988) using G*power3.1. (Faul et al., 2007). Finally, the subjects comprised 36 healthy adults (17 men, 19 women, average age: 23.56 ± 4.61 years old, schooling history: 16.39 ± 1.10 years). The average score of the analyzed subjects on the handedness test was 0.90 ± 0.25.

## Experimental Tasks

### Stimulation and Condition of a Single Task

This study was performed in accordance with the approval of Tsukuba faculty of human sciences (Permit number: 29–134). All subjects obtained written informed consent in accordance with the Declaration of Helsinki. We excerpted 18 sentences from a workbook for the Japanese Language Proficiency Test [NPO Research Institute for Japanese Language Education (1995)]. The average stimuli of the sentences were 154.83 ± 4.24 morae, and they were displayed horizontally in the center of a computer monitor with a character size of approximately 1 cm × 1 cm.

In the experiment, the subjects were asked to read aloud the sentences that appeared on the monitor placed 60 cm in front of their eyes. The subject’s voice was captured during the task using a microphone (SHURE SM58) placed within 15 cm of his/her mouth, and this was provided as feedback through closed headphones (SONY MDR-7506) worn by the speaker over both ears through a mixer (MACIE 802VLZ4) and effector (LEXICON MX300). The headphones were used to prevent the subject’s voice from coming outside the headphones and overlap with the subject’s voice recorded by the microphone and played on the headphones. In addition, the subject’s voice was recorded on an integrated circuit (IC) recorder (OLYMPUS Voice Trek V-863) with a microphone also placed within 15 cm of his/her mouth.

**Figure 1:**
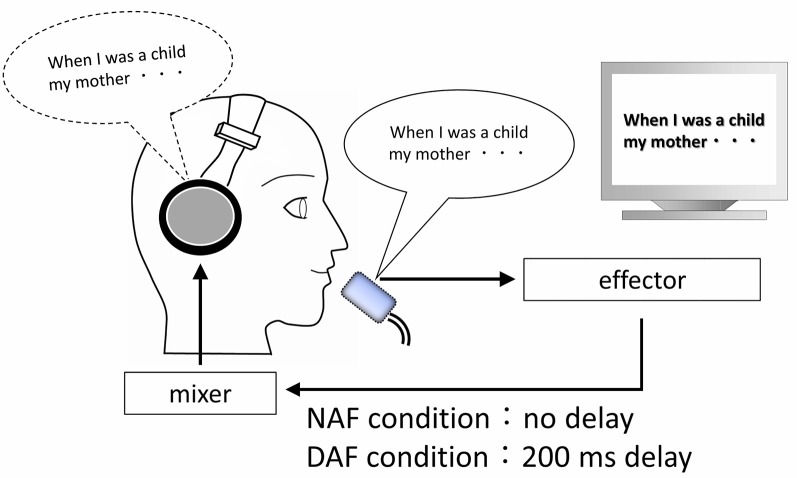
Conditions of voice feedback.

During the single task (reading-aloud task), the auditory feedback of the subject’s voice was given under NAF or DAF (Figure 1). Under NAF, the subject’s voice was feedback to the subject immediately with almost no delay (in real time), while he/she was reading the sentences aloud. In contrast to NAF, the subject’s voice was provided as feedback to the subject with a delay of 200 ms by using an effector under single DAF. One reading-out-loud task lasted for 15 s per trial, and the subjects were asked to perform three trials under each condition with different reading stimuli.

The delay time was set to 200 ms in the present study because the frequency of nonfluent speech was high in this delay time compared to other delay times (Fairbanks, 1955; MacKay, 1968; Siegel et al., 1980; Stuart et al., 2002).

### Stimulation and Condition of Dual Tasks

**Figure 2:**
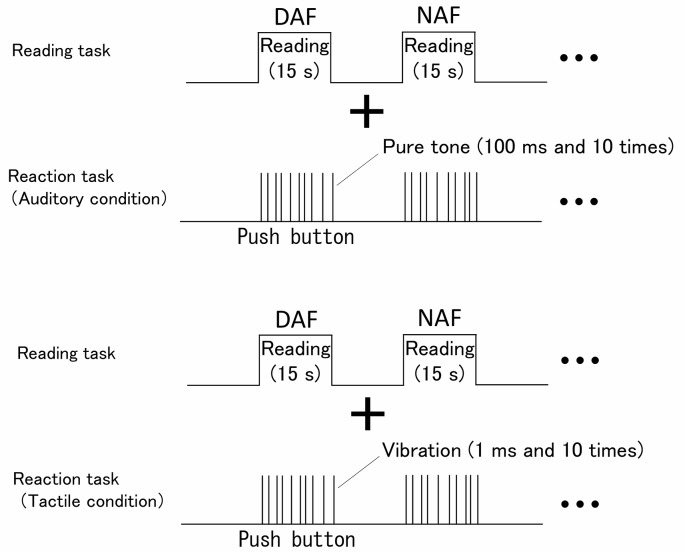
Conditions of dual tasks (auditory condition and tactile condition).

During the dual tasks, the subjects were asked to react to tactile or auditory stimuli while reading aloud sentences (Figure 2). In the tactile condition, which requires cross-modal attention allocation, the rod-shaped solenoid vibratory stimulation element of a 1-CH tactile stimulation device (UCHIDA FB-1200LP) was attached to the subject’s left index finger by using surgical tape, and vibration stimuli were presented through the element. The stimuli were presented pseudorandomly 10 times in one trial with a Stimulus Onset Asynchrony (SOA) of 600 ms to 2,400 ms. We asked the subject to prioritize reading the sentences when it was displayed on the monitor and to press a button on the keyboard by using right index finger as quickly as possible every time if vibration stimulation was provided to the left index finger while reading. In the auditory condition, which requires unimodal attention allocation, a pure tone (1,000 Hz) was presented for 100 ms through a mixer and effector to the subject from the headphones. The pure tone was presented 10 times pseudorandomly in one trial with an SOA of 600–2,400 ms. We asked the subject to prioritize reading the sentence when it was displayed on the monitor, and to press a button as quickly as possible every time if the pure tone was presented while continuing reading aloud.

The dual tasks involving tactile or auditory stimulation were both performed under the conditions of NAF and DAF. The same sentences of the single task were used. Each dual task was performed thrice with one trial lasting 15 s. E-Prime 2.0, a psychological experiment software, was used for presenting and controlling tactile and auditory stimulation.

## Procedure

We investigated the relationship between feedback and attention under six conditions: single NAF condition, single DAF condition, tactile NAF condition, tactile DAF condition, auditory NAF condition, and auditory DAF condition. Each of these six tasks were performed thrice, while alternating between NAF and DAF [e.g., tactile NAF condition (15 s) ⇒ tactile DAF condition ⇒ tactile NAF condition ⇒ tactile DAF condition ⇒ tactile NAF condition ⇒ tactile DAF condition ⇒ single NAF condition ⇒ single DAF condition …]. The sentences, the order of stimulus modality (single/tactile/auditory), and the order of feedback condition (NAF → DAF or DAF → NAF) were counterbalanced among the subjects. A fixation point “+” was presented every 5 s before and after each trial, and the subjects were asked to gaze at the center of the point when presented.

## Analysis

We calculated the reaction time that elapsed between the presentation of stimulation and button press (within 600 ms) and the omission error rate for each subject. For the reaction time and omission error rate, we performed a 2 (NAF and DAF)-by-2 (tactile and auditory) intrasubject two-way analysis of variance (ANOVA) with Bonferonni correction. As we have analyzed the data twice (when the sample size was 22 and 36), an alpha level of 0.025 was set for the analysis in order to maintain an experiment-wise error rate of 5%.

In addition, a professional teacher calculated the nonfluency rate of each subject under each condition by calculating the proportion of nonfluent speech (nonfluent phrase/all phrases×100). Phrase is a unit that contains one independent word. Based on the work by Sakai et al. (2009), we divided nonfluency into nine classifications: (1) sound repetition (w, w, when I …); (2) part-word repetition (whe, when, I…); (3) word and phrase repetition (when I, when I was…); (4) prolongation (wh-en I); (5) break (when…I was…); (6) distortion; (7) syllable repetition (when, when I was …); (8) revision (where, when I was…); and (9) error (where I was…). To ensure the measurement reliability of the nonfluency ratings, the nonfluency rate was recalculated by the same professional teacher 1 month after first assessment, and 10% segments were selected at random (intra-rater reliability). Inter-rater reliability was determined by a second professional teacher assessing nonfluent part for 10% of the sample selected at random. Then, an intraclass correlation coefficient analysis was conducted. We performed a 2 (NAF and DAF)-by-3 (single, tactile, and auditory) intrasubject two-way ANOVA to compare the nonfluency rate among different conditions. We also calculated the DAF effect (the difference in the nonfluency rate between the single NAF condition and single DAF condition) to investigate the susceptibility to DAF and the relationship between increased/decreased fluency in each modality. Furthermore, we calculated the Pearson correlation coefficient between the increased/decreased fluency rate (tactile DAF condition minus single DAF condition) under the tactile condition and DAF effect, and the coefficient between the increased/decreased fluency rate (auditory DAF condition minus single DAF condition) under the auditory condition and DAF effect. SPSS version 25.0 was used for statistical analysis.

## Results

### Reaction Time and Omission Error Rate

The reaction time and omission error rate under the tactile and auditory conditions are listed in Table 1.

**Table 1:** Reaction time and omission error rate under tactile and auditory conditions.

	Reaction time (ms)		Omission error rate (%)	
	Tactile	Auditory	Tactile	Auditory
NAF	260.9 ± 58.7	312.0 ± 69.5	6.3 ± 5.7	8.7 ± 7.4
DAF	311.6 ± 85.3	365.6 ± 92.0	15.1 ± 12.2	16.7 ± 12.1

*NAF, nonaltered auditory feedback; DAF, delayed auditory feedback*.

The mean of the reaction time in the tactile condition was 260.9 ± 58.7 ms under the NAF condition and 311.6 ± 85.3 ms under the DAF condition. The mean reaction time in the auditory condition was 312.0 ± 69.5 ms under the NAF condition and 365.6 ± 92.0 ms under the DAF condition. There was a significant main effect of the modality (tactile/auditory; *F*_(1,35)_ = 42.2, *p* < 0.005, $\eta_{\text{G}}^{2}$ = 0.10), and the reaction time was significantly shorter under the tactile condition than under the auditory condition. The feedback (NAF/DAF) also showed significant main effects (*F*_(1,35)_ = 43.12, *p* < 0.005, $\eta_{\text{G}}^{2}$ = 0.11), and the reaction time was significantly shorter under NAF than under DAF. No significant interaction between the modalities and feedback was observed (*F*_(1,35)_ = 0.55, *p* = 0.82, $\eta_{\text{G}}^{2}$ = 0.00).

The mean of the omission error rate in the tactile condition was 6.3 ± 5.7% under the NAF condition and 15.1 ± 12.2% under the DAF condition. The mean of the omission error rate in the auditory condition was 8.7 ± 7.5% under the NAF condition and 16.7 ± 12.1% under the DAF condition. There were significant main effects of the feedback (NAF/DAF; *F*_(1,35)_ = 30.3, *p* < 0.005, $\eta_{\text{G}}^{2}$ = 0.16), and the omission error rate was significantly higher under DAF than under NAF. However, the omission error rate of the modalities (tactile/auditory) showed no main effects (*F*_(1,35)_ = 2.5, *p* = 0.13, $\eta_{\text{G}}^{2}$ = 0.01) and interaction between the modalities and feedback (*F*_(1,35)_ = 0.3, *p* = 0.61, $\eta_{\text{G}}^{2}$ = 0.00).

### Nonfluency Rate

**Figure 3:**
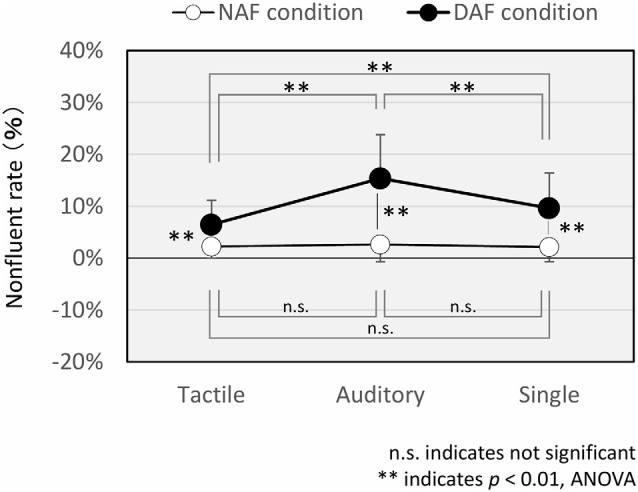
Nonfluent rate in the single and dual tasks. Error bar means standard deviation.

The nonfluency rates in the single task, dual tactile task, and dual auditory task are shown in Figure 3. With respect to nonfluent judgment, we obtained sufficient reliability of assessment (intra-rater reliability: 0.98 and inter-rater reliability: 0.72).

The mean nonfluency rate in the single task was 2.1 ± 2.9% under the NAF condition and 9.6 ± 6.7% under the DAF condition. The mean nonfluency rate in the tactile condition was 2.3 ± 2.2% under the NAF condition and 6.5 ± 4.7% under the DAF condition. The mean nonfluency rate in the auditory condition was 2.6 ± 3.4% under the NAF condition and 15.4 ± 8.4% under the DAF condition. There were significant main effects of the feedback (NAF/DAF: *F*_(1,35)_ = 83.56, *p* < 0.005, $\eta_{\text{G}}^{2}$ = 0.38) and modalities (single/tactile/auditory: *F*_(2,70)_ = 38.84, *p* < 0.005, $\eta_{\text{G}}^{2}$ = 0.12). The nonfluency rate was significantly higher under the DAF condition than under the NAF condition. Moreover, there was a significant interaction between the feedback and modalities (*F*_(2,70)_ = 44.56, *p* < 0.005, $\eta_{\text{G}}^{2}$ = 0.10). We performed a main-effect test, and there was no significant difference in the nonfluency rate among the different NAF conditions (single/tactile/auditory; *F*_(2,70)_ = 0.63, *p* = 0.53, $\eta_{\text{G}}^{2}$ = 0.01). In contrast, the nonfluency rate of the DAF condition was significantly lower under the tactile condition than under the single condition (*t*_(35)_ = 3.56, *p* < 0.005, *d* = 0.54). However, it was significantly higher under the auditory condition than under the single condition (*t*_(35)_ = 7.46, *p* < 0.005, *d* = 0.75). The nonfluency rate was significantly higher under the auditory condition than under the tactile condition (*t*_(35)_ = 9.24, *p* < 0.005, *d* = 1.30). In addition, there was a significant difference between NAF and DAF conditions for the single task and dual tasks with tactile and auditory stimulation (tactile: *F*_(1,35)_ = 35.48, *p* < 0.005, $\eta_{\text{G}}^{2}$ = 0.25; auditory: *F*_(1,35)_ = 98.02, *p* < 0.005, $\eta_{\text{G}}^{2}$ = 0.50; single: *F*_(1,35)_ = 53.07, *p* < 0.005, $\eta_{\text{G}}^{2}$ = 0.34). The nonfluency rate under the DAF condition was higher than under NAF conditions in all tasks (single/tactile/auditory).

### Correlation With DAF Effect

**Figure 4:**
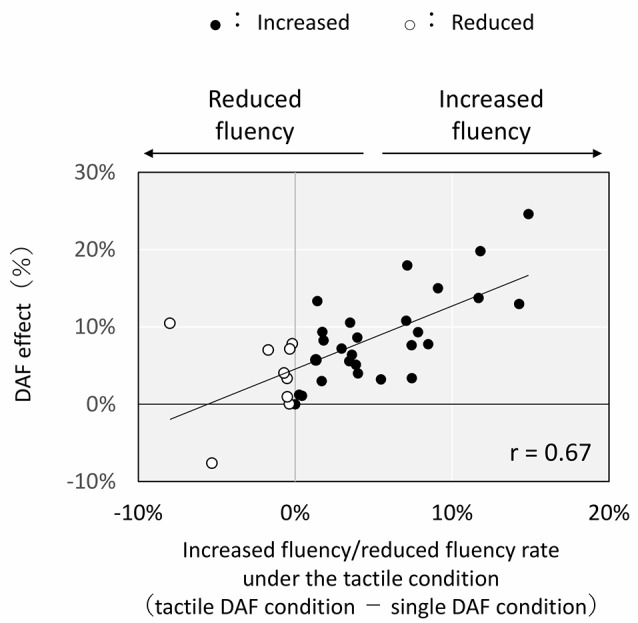
Correlation between delayed auditory feedback (DAF) effect and increased fluency/reduced fluency rate under the tactile condition. Each plot shows the result for each subject. DAF effect: difference in the nonfluency rate between the single normal auditory feedback (NAF) condition and single DAF condition. Increased fluency: nonfluency rate under the tactile DAF condition < nonfluency rate under the single DAF condition. Reduced fluency: nonfluency rate under the tactile DAF condition > nonfluency rate under the single DAF condition.

**Figure 5:**
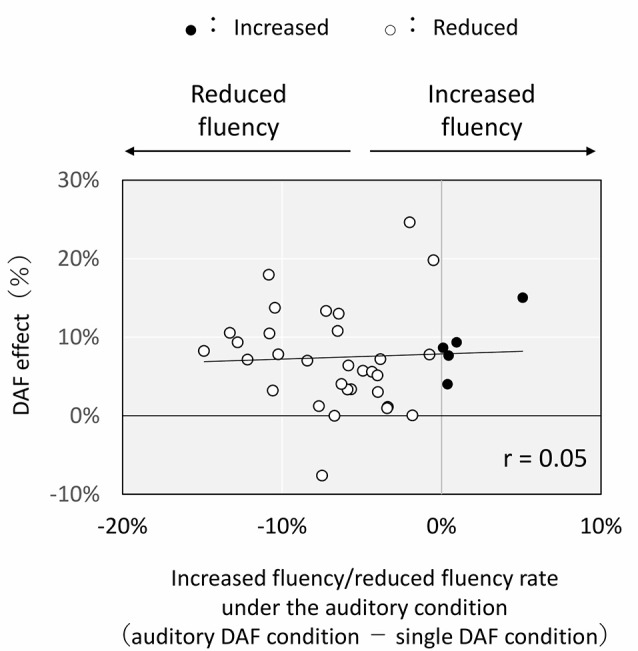
Correlation between DAF effect and increased fluency/reduced fluency rate under the auditory condition. Each plot shows the results for each subject. DAF effect: difference in the nonfluency rate between the single NAF condition and single DAF condition. Increased fluency: nonfluency rate under the auditory DAF condition < nonfluency rate under the single DAF condition. Reduced fluency: nonfluency rate under the auditory DAF condition > nonfluency rate under the single DAF condition.

There was a significant positive correlation between the DAF effect and the increased/decreased fluency rate (tactile DAF condition–single DAF condition) under the tactile condition (Figure 4; *r* = 0.67, *p* < 0.005). In contrast, there was no correlation between the DAF effect and the increased/decreased fluency rate (auditory DAF condition–single DAF condition) under the auditory condition (Figure 5; *r* = 0.05).

## Discussion

### Relationship Between Delayed Auditory Feedback and Attention

This study investigated the relationship between nonfluent speech and auditory attention to vocal feedback by using reading tasks. In the single task, the subjects had to read aloud sentences by actively allocating attention to the sentences (task-relevant stimulus). DAF (task-irrelevant stimulus) deviates the subjects from their internal prediction signal generated in parallel with the movements of the subject’s speech, and it is considered to be a highly salient stimulus. In particular, auditory stimuli are reported to be more likely to capture attention than visual stimuli (Berti and Schröger, 2001; Boll and Berti, 2009; Leiva et al., 2015). We presumed that speech became nonfluent because attention, which was originally allocated to the reading task, was being captured by the delayed voice (deviant, task-irreverent stimulus).

One possible explanation for the occurrence of nonfluency under DAF is that the time lag between auditory feedback and internal signal of that efferent motor control that predicts the auditory feedback of the motor command is influenced under DAF (Howell, 2004; Max et al., 2004). The auditory feedback-based online correction to minimize the internal signal and feedback information may contribute to speech nonfluency (Max et al., 2004). Attention capture could be one of the explanations of the DAF effect since there could be a room for attention system in the feedback control, and we should further elaborate the hypothesis. Though other feedback parameters can change (e.g., frequency-altered auditory feedback), we believe that this attention capture effect that could cause disfluent speech is specific to the delay of own voice. Because the phenomenon of disfluent speech was only found under DAF, and not under any other conditions; e.g., frequently altered auditory feedback (Natke et al., 2001).

### Dual Tasks and Attention Capture by Delayed Voice

We also investigated the relationship between auditory attention captured by a delayed voice and nonfluent speech using dual tasks. In a dual task, the subjects are asked to perform a secondary task in addition to the primary task. This causes limited attentional resources to be allocated to each task (Kahneman, 1973). The tactile DAF condition is a cross-modal condition wherein the secondary task (reacting to tactile vibration) is irrelevant to the primary task (reading aloud under DAF). In contrast, the auditory DAF condition is a unimodal condition wherein the secondary task (reacting to auditory tone) is relevant to the primary task (reading aloud under DAF). The correct answer rate for the reaction task was over 80% under all conditions. Hence, in the dual tasks, the subjects continuously allocated attention to not only the sentences but also the target of the secondary task under each modality. This seems apparently inconsistent because, if the DAF increases the auditory attention of the subject and the attention of the auditory feedback involved an auditory task performance, performance parameters such as reaction time or the omission error rate could improve. We assumed that attention allocation under the DAF condition could not be generalized in the context of attention system. Under DAF, their fluency indeed decreased. It was also found that the performance under the DAF condition was poorer than under the NAF condition, and a significant interaction did not occur between the modality (tactile/auditory) and feedback (NAF/DAF) for both the reaction time and omission error rate. Thus, the decrease in performance under DAF is not limited in the auditory task.

Regarding the nonfluency rate in the primary task, there was no significant difference between the conditions under NAF. However, there were significant differences between the conditions under DAF. The nonfluency rate in the dual task with auditory stimulation was significantly higher than that in the single task, while the nonfluency rate in the dual task with tactile stimulation was significantly lower than that in the single task. It is assumed that, in the auditory task under DAF condition, the subjects allocated attention to not only the sentences but also the auditory modality in order to promptly react when the secondary target arrived. The subjects seem to constantly prepare for the detection of and a reaction to the next target when the target was successively presented, and the delayed voice was superimposed within such task sets. A predetermined task set could cause attention to be captured by the subsequent distracting stimuli (Eimer and Kiss, 2008). Thus, we suggested that the nonfluency rate could increase in the auditor task because of the enhanced attention capture to a delayed voice that was designed for the detection of the auditory target.

This leads to the question of what attentional processes took place under the tactile condition, where nonfluent speech decreased compared to that in the single task. Compared with the auditory condition, the subjects actively allocate attention to the visual-modality sentences (primary task) and the tactile-modality target (secondary and cross-modal task). Attention allocation to auditory stimuli decreased when attention was directed to sequences of tactile stimulus (Marsja et al., 2018). Neuroimaging studies also revealed that the activity of the auditory cortex is attenuated when visual stimuli are presented, while the activity of the visual cortex is attenuated when tactile or auditory stimuli are presented (Laurienti et al., 2002; Merabet et al., 2007). We suggested that, under the tactile condition, the auditory perception of the delayed voice could be inhibited, and attention was captured by the delayed voice to a lesser degree than under the other conditions of DAF. This is because attention was allocated to visual and tactile sensory input. The distracting effect of the delayed voice was attenuated, and nonfluent speech was reduced as a result. Through our findings, we could interpret that nonfluent speech occurs depending on the deviance of the disruption stimulus. That is, task-irrelevant and unimodal stimulus divert attention from the speech, thus resulting in less nonfluency and vice versa.

There are individual differences in the reduction of nonfluent speech. As shown in Figure 4, subjects who had a larger DAF effect (i.e., speech disfluency frequently occurs under single DAF condition rather than under single NAF condition) have a tendency to decrease their disfluency under the tactile DAF condition. We assume that disfluent speech resulted in the present study because of inappropriate allocation of attention to delayed voice. The tactile task made the subjects who exhibited a larger DAF effect distract their attention into the tactile (cross-modal) stimulus. Our results suggest that this could increase speech fluency.

In contrast, no significant difference was observed in the nonfluency rate among the different conditions under NAF. The relationship between attention allocation and nonfluent speech was only found under DAF. It is supposed that the subject could allocate sufficient attention to secondary (tactile/auditory) tasks.

The present study showed that attention capture by delayed own voice could result in nonfluent speech. However, it remains unclear if attention capture by delayed own voice is involved in matching or the subsequent movement correction process. A study that used event-related potential (ERP) showed that this matching and movement correction are different processes (Rietdijk et al., 2014). Further studies investigating the impact of attention capture on nonfluent speech using neuroimaging approaches are required. Please note that this study used only DAF at a delay of 200 ms. As our findings regarding nonfluency occurrence by auditory attention capture is at a preliminary level rather than an origin causality, and further studies including conditions such as changing the delay time or frequency of the auditory feedback are needed to elaborate our findings.

## Data Availability Statement

The datasets generated and/or analyzed for this study are not publicly available due to specifications on data availability within the ethics approval. Data are, however, available from the corresponding author upon reasonable request and with the permission of the ethics committee.

## Ethics Statement

The studies involving human participants were reviewed and approved by Tsukuba faculty of human sciences, University of Tsukuba, Japan. The patients/participants provided their written informed consent to participate in this study.

## Author Contributions

All authors contributed to the conception or design of the work or the acquisition, analysis, or interpretation of data for the work. OI and DI contributed to the writing of the manuscript. SM contributed to manuscript revision and read and approved the submitted version.

## Conflict of Interest

The authors declare that the research was conducted in the absence of any commercial or financial relationships that could be construed as a potential conflict of interest.
